# Risk factors for myocardial infarction among low socioeconomic status South Indian population

**DOI:** 10.1186/1758-5996-2-32

**Published:** 2010-05-26

**Authors:** Ramachandran Meenakshisundaram, Dipti Agarwal, Chinnaswamy Rajendiran, Ponniah Thirumalaikolundusubramanian

**Affiliations:** 1 Institute of Internal Medicine, Madras Medical College, Chennai, India; 2 Medicine, J.J.M Medical College, Devangere, India; 3 Department of Medicine, Chennai Medical College Hospital & Research Center, Irungalur, Trichy, India

## Abstract

**Background:**

As longevity increases, cases of myocardial infarction (MI) are likely to be more. Cardiovascular disease (CVD) is a major global health problem reaching epidemic proportions in the Indian subcontinent, also among low socio-economic status (SES) and thin individuals.

**Objectives:**

The present study was undertaken to elicit risk factors for MI among low SES Southern Indians and to find out its association with body mass index (BMI).

**Materials and methods:**

A case-control study of patients with MI matched against healthy control subjects was carried out in a tertiary care teaching hospital. Standard methods were followed to elicit risk factors and BMI. Chi-square and Fishers exact test for categorical versus categorical, to show relationship with risk factors were analyzed.

**Results:**

A total of 949 patients (male (M) = 692 and post menopausal female (F) = 257) and 611 age and sex matched healthy controls were included. In our study, BMI was below 23 in 48.2% of patients and below 21 in 22.5%. The risk of developing MI was significantly more in males (odds ratio (OR) = 3.3, 95% confidence interval (C.I.) = 2.69-4.13), among females with post-menopausal duration (PMD) of more than or equal to 3 years (OR = 9.27, 95% C.I. = 6.36-13.50) and in those with BMI less than 23 with one or other risk factors (P = 0.002, OR = 1.38, 95% C.I. = 1.13-1.70).

**Conclusion:**

BMI cannot be considered as a lone independent risk factor, as the study population had low BMI but had one or more modifiable risk factors. It would be advisable to keep BMI at least 21 kg/m^2 ^for screening program. Health education on life style modification and programs to diagnose and control diabetes and hypertension have to be initiated at community level in order to reduce the occurrence.

## Introduction

Cardiovascular disease (CVD) is a major global health problem reaching epidemic proportions in the Indian subcontinent [1] and low and middle income countries, accounting for 78% of all deaths [2]. High risk of CVD has been reported among South Asians, regardless of whether they live overseas or in their native lands [2,3]. Presence of conventional risk factors such as smoking, diabetes mellitus, hypertension and dyslipidemia are clearly associated with coronary artery disease (CAD) among them. Even though, many of them were non-obese [4,5]. However, obesity, as defined by body mass index (BMI) of ≥30 kg/m^2^, is less prevalent in South Asians [6], which is also considered as an important risk factor for CAD. Some investigators have reported lower BMI cutoffs for Indians based on visceral fat [7]. In Indians, conventional cutoff limit of BMI [8] might not define overweight and obesity optimally, because of their higher percentage of body fat and less lean mass [9]. Hence, World Health Organization (WHO) has redefined overweight (BMI ≥ 23 kg/m^2^) and obesity (BMI ≥ 25 kg/m^2^) for South Asians [10], based on the preliminary data, which are under debate [11]. The present study was undertaken to elicit the pattern of selected risk factors for myocardial infarction (MI) among South Indians belonging to low socioeconomic status (SES) and their association with BMI.

## Materials and methods

### Study Settings and Designs

This prospective case control study was undertaken in Madras Medical College, the pioneer tertiary referral and teaching center of Southern India located at Chennai. This study was conducted according to the guidelines laid down in the Declaration of Helsinki and all procedures involving human subjects/patients were approved by the institutional ethical committee. A witnessed and formally recorded verbal informed consent was obtained from all subjects/patients. We have enrolled 949 cases and 611 age and sex matched controls over a period of 18 months and patient enrollment was consecutive. Cases confirmed by electrocardiogram and diagnostic enzyme changes, were alone included. Subject groups with previous history of MI, stroke, heart disease, liver disease, blood disorders, type 1 diabetes, and other co-morbid illnesses were excluded. Also, patients with risk factors other than smoking, diabetes, hypertension, and dyslipidemia were excluded, since they are not major concern in Indian population [12]. All subject groups belonged to low SES according to modified Kuppusamy rating scale, which included education, occupation and monthly income of the family with total score ≤10 [13] and all women were in post-menopausal status.

### Definitions

Patients with persistent elevation of blood pressure (≥140/90 mmHg) or who were on anti-hypertensive drugs were classified as hypertensives [14]. Dyslipidemia was defined as hypercholesterolemia, hypertriglyceridemia, high low density lipoprotein (LDL) and low high density lipoprotein (HDL), according to the criteria of National Cholesterol Education Program, Adult Treatment Panel III [15] or patients whoever on drugs to lower cholesterol. Diabetes in our dataset were only type 2 diabetes and was defined using the American Diabetes Association criteria of fasting plasma glucose >125 mg/dl [16] or patients taking hypoglycemic drugs. Smokers in our dataset had pack year duration ≥20 years. Post-menopausal duration (PMD) is defined as the duration between onset of menopause and MI.

### Statistical Methods

Demographic and clinical characteristics such as age, sex, smoking behavior, diet, physical activity, weight, height, PMD, blood pressure, plasma sugar, lipid profile were recorded in the proforma, and entered in a Microsoft office excel (2003) sheet. Data were sub-divided into various categories such as isolated risk factors viz., smoking, hypertension, diabetes, and dyslipidemia only or a combination of them or no risk factors. Also, females were classified into 2 categories; PMD of less than 3 and ≥3 years. Descriptive analysis was employed for all independent and dependent variables. Chi-square and Fishers exact test for categorical versus categorical and other data analysis were carried out to elicit relationship with risk factors by using Statistical Package, STATPAGES [17].

## Results

A total (T) of 949 patients (male (M) = 692 and post menopausal female (F) = 257) with their ages varying from 45 to 64 years and 611 healthy control subjects (age and sex matched) were enrolled. Their SES and physical activity were low, but dietary content of carbohydrate (80% calorie) and salt level (10-12 grams/day) were high. In our dataset, males (odds ratio (OR) = 3.3, 95% confidence interval (C.I.) = 2.69-4.13) had higher risk than females. Interestingly, among females the risk was significantly more in those with PMD ≥ 3 years (OR = 9.27, 95% C.I. = 6.36-13.50). None of the females were smokers due to their cultural habits [18]. Distribution of risk factors among MI is shown in Table 1 and prevalence of subject groups is shown in Table 2. Significant number of patients (434) with BMI < 23 kg/m^2 ^along with one or other risk factors had MI (Chi-square = 9.599, P = 0.002, OR = 1.38, 95% C.I. = 1.13-1.70). None had any medical checkup earlier. Isolated smoking and hypertension among non-obese individuals (BMI < 23), isolated hypertension and diabetes independent of BMI, have significant risk for MI. The statistical associations between different groups of MI are shown in Table 3.

**Table 1:** Distribution of risk factors in cases as per BMI.

BMI **in kg/m**^**2**^	No. of patients			Without risk factor			Smoking only			Hypertension only			Diabetes only			Dyslipidemia only			More than 1 risk factor		
	**M**	**F**	**T**	**M**	**F**	**T**	**M**	**F**	**T**	**M**	**F**	**T**	**M**	**F**	**T**	**M**	**F**	**T**	**M**	**F**	**T**

15 - 16.9 16.9	20	2	22	1	0	1	7	0	7	0	0	0	3	0	3	0	0	0	9	2	11

17 - 18.9	50	11	61	0	0	0	21	0	21	5	2	7	3	1	4	0	1	1	21	7	28

19 - 20.9	104	27	131	5	4	9	31	0	31	9	2	11	10	6	16	0	0	0	49	15	64

21 - 22.9	181	63	244	11	3	14	39	0	39	31	11	42	13	5	18	0	0	0	87	44	131

23 - 24.9	210	88	298	6	4	10	29	0	29	12	7	19	22	21	43	2	1	3	139	55	194

25 - 26.9	90	37	127	1	2	3	5	0	5	4	3	7	15	6	21	0	0	0	65	26	91

27 - 28.9	31	21	52	0	0	0	0	0	0	0	1	1	1	0	1	1	0	1	29	20	49

29 - 30.9	5	5	10	0	0	0	0	0	0	0	1	1	0	0	0	0	0	0	5	4	9

31 - 32.9	1	3	4	0	0	0	0	0	0	0	0	0	0	0	0	0	0	0	1	3	4

																					

**Total**	692	257	949	24	13	37	132	0	132	61	27	88	67	39	106	3	2	5	405	176	581

BMI = Body mass index, M = Male, F = Female, T = Total, No. = Number, Only = Isolated without any other risk factors

**Table 2:** Prevalence of Subject Groups in relation to Selected Factors.

Factors	Control Subjects, N (%)	Cases, N (%)
Blood pressure		
≥140/90 mmHg	81 (13)	460 (48)
<140/90 mmHg	530 (87)	489 (52)

Plasma Glucose level		
>125 mg/dl	71 (12)	465 (49)
≤125 mg/dl	540 (88)	484 (51)

Dyslipidemia	47 (8)	185 (19)

Smoking	114 (19)	484 (51)

N = Number, % = Percentage

**Table 3:** Odds Ratios (95% Confidence Intervals) for Myocardial Infarction According to BMI & Independent of BMI.

Risk Factors	**BMI < 23 kg/m**^**2**^	**BMI 23-24.9 kg/m**^**2**^	**BMI **≥ **25 kg/m**^**2**^
No risk factor	0.05 (0.03-0.08)	0.01 (0.007-0.02)	0.018 (0.006-0.06)

Smoking only	1.74 (1.17-2.59)*	0.36 (0.22-0.59)	0.52 (0.17-1.55)

Hypertension only	2.94 (1.65-5.22)*	0.65 (0.33-1.28)	0.67 (0.28-1.62)

Diabetes only	4.16 (1.95-8.84)*	1.61 (1.18-2.98)*	3.35 (1.30-8.61)*

Dyslipidemia only	0.08 (0.01-0.49)	0.09 (0.03-0.29)	0.23 (0.03-1.58)

Smoking	5.67 (4.03-7.97)*	2.04 (1.44-2.89)*	9.16 (4.69-17.89)*

Hypertension	9.15 (6.09-13.73)*	2.85 (1.89-4.29)*	8.62 (5.06-14.68)*

Diabetes	14.39 (9.04-22.91)*	3.64 (2.42-5.46)*	14.33 (7.91-25.98)*

Dyslipidemia	3.88 (2.19-6.90)*	1.62 (1.05-2.59)*	5.96 (2.74-12.98)*

**Risk Factors**	**Overall (Independent of BMI)**		

Smoking only	0.97 (0.73-1.30)		

Hypertension only	1.46 (1.11-2.15)*		

Diabetes only	2.72 (1.77-4.19)*		

Dyslipidemia only	0.11 (0.04-2.78)		

Smoking	4.59 (3.57-5.67)*		

Hypertension	9.59 (7.34-12.52)*		

Diabetes	7.87 (5.95-10.41)*		

Dyslipidemia	2.98 (2.13-4.97)*		

BMI = Body mass index, Only = Isolated without any other risk factors, * = P < 0.05, significant.

## Discussion

This is the first reported case-control study aimed to analyze the risk factors for MI among low SES Southern Indians. In the present study, it was observed that low BMI, smoking, diabetes, hypertension and dyslipidemia were found to be an independent risk factor for MI, as observed in an earlier study [19]. All risk factors were significantly more in both sexes when compared cases against healthy control subjects. However, there was considerable heterogeneity in the prevalence among them; males have higher risk than females [20]. Though vegetarianism being common among Indians, most of them consume high carbohydrate, high salt and low fiber diet such as green leafy vegetables and fruits [20], which contribute to increase in triglyceride, blood pressure and plasma sugar, and also, have higher risk for MI [21]. The daily moderate physical activity of brisk walking for 45 minutes was associated with 50% reduction in risk for MI [22], which was not regularly practiced in our subject groups. Our findings of increased occurrence of MI among low SES, accord with Gupta et al., and the prevalence of CAD was inversely related to the level of education and income [23].

In the current study, 77.6% of population with BMI below 25 had MI and one or other risk factors [24]. Also, multiple cardiovascular risk factors were seen in the patients with BMI < 25 kg/m^2 ^[4,24]. Vikram et al. [24], concluded that non-obese Indians with BMI < 25 kg/m^2 ^and normal waist circumference have higher cardiovascular risk. Interestingly, low BMI is also found to be a risk factor for CAD [25-27]. This is due to impaired endothelium-dependant vasodilation through increased oxidative stress, leading to the reduced bioavailability of nitric oxide. Endothelial dysfunction is an early finding in the arteriosclerosis and increases the incidence of CAD. Further, low birth weight is considered to be a risk factor for MI, in later life or for premature MI (less than 50 years of age) [28,29]. From this, it is concluded that low BMI may be a risk factor for MI in later life.

Smoking, an established risk factor for MI [19,30] is associated with endothelial dysfunction and can precipitate coronary spasm [31]. Cases and control subjects had smoked or were current smokers of cigarettes/beedis. Beedis are the forms of local type of tobacco from temburni leaf (Diospyros melanoxylon) and more lethal than cigarettes. There was an increased risk among cases, who smoked beedis [30]. In our dataset, significant number of smokers with BMI < 23 kg/m^2 ^(OR = 1.74, 95% C.I. = 1.17-2.59) had MI, but this significance was not observed among smokers with BMI ≥ 23 kg/m^2^. All forms of tobacco produce free radicals that deplete antioxidants and cause oxidative damage to DNA, proteins and lipids [32]. Antioxidants- rich foods such as vegetables and foods may help to reduce the oxidative stress caused by tobacco [33], are usually lacking in the diet of low SES [34] who also have low BMI thus pushing them to develop MI. Smoking is inversely associated with BMI at lower level of education (low SES) and positively associated with BMI at higher level of education [35].

In our study, diabetes is an important risk factor for MI, even in patients with BMI < 23 kg/m^2 ^(OR = 4.16, 95% C.I. = 1.95-8.84). Also, isolated diabetes (OR = 2.72, 95% C.I. = 1.77-4.19) and diabetes with one or other risk factors (OR = 7.87, 95% C.I. = 5.95-10.41) were found to have an association with MI. As diabetes is an independent risk factor for MI, it is considered to be an equivalent to CAD [36]. Abnormalities of glucose metabolism are common in Indians and often occur without significant obesity [37]. Ramachandran et al., showed the prevalence of diabetes and impaired glucose tolerance (IGT) in Indians were 12 and 14% respectively, and which were independent of gender [38]. Both diabetes and IGT are associated with increased risk of CAD even within the ranges considered normal [39]. Approximately, 80% of deaths in diabetic patients are attributable to CAD [40], which in turn is highly correlated with dyslipidemia [40].

In our dataset, isolated hypertension (OR = 1.46, 95% C.I. = 1.11-2.15) and hypertension with one or other risk factors (OR = 9.59, 95% C.I. = 7.34-12.52) were significantly associated with MI. Hypertension is closely correlated with salt intake, alcohol intake and obesity. The mean salt intake among Indians is 8.5 grams/day [41], which is higher than the recommended level. In people with normal or high blood pressure, dietary salt restriction reduces blood pressure at 6-12 months [42] and weight reduction of 9 kg, can lower systolic blood pressure by 6 mm Hg and diastolic blood pressure by 3 mm Hg in hypertensive patients [43]. Interestingly, significant number of patients with Hypertension and BMI < 23 kg/m^2 ^(OR = 2.94, 95% C.I. = 1.65-5.22) had MI, but this observation was not seen in those with BMI ≥ 23 kg/m^2^. This could be explained by the fact that low BMI might have induced endothelial impairment in such populations [27].

Though lipid abnormalities are widely accepted as risk factor for MI, less often noticed among Indian population [30] especially among low SES group. Isolated dyslipidemia did not contribute to the risk (OR = 0.11, 95% C.I = 0.04-2.78) in our dataset. However, dyslipidemia in association with one or other risk factors contributed significantly to MI (OR = 2.98, 95% C.I. = 2.13-4.97). Risk of MI is equal among post-menopausal women and men, an established fact. Interestingly, females with PMD ≥ 3 years have higher risk than females with PMD < 3 years. From this, we conclude that longer the PMD, greater the risk for MI. Hence, all post-menopausal women may be counseled towards the risk and screening measures for MI.

## Conclusion

India is currently in the middle of MI epidemic that was initially observed among immigrant Indians. In the present report, higher prevalence of risk factors and MI were seen in patients even with BMI < 23 kg/m^2^. These observations clearly support the recent WHO initiatives and its debates, to revise the normal limits of BMI. Hence, it would be advisable to redefine the BMI ≥ 23 kg/m^2 ^as overweight and BMI ≥ 25 kg/m^2 ^as obese for South East Asians. Screening measures for risk factors of MI may be initiated for people with BMI ≥ 21 kg/m^2^. All Post-menopausal women may be advised to start their screening measures immediately after menopause. All clinicians should search routinely for risk factors among every case and counsel the identified victims. Moreover, recognition and adoption of BMI cutoffs represent a major step forward in redefining the risk stratification among Indians.

## Disclosures

Financial disclosure - Nil

Conflict of interest - Nil

## Authors' contributions

RM carried out study design, study protocol, sample collection, statistical analysis, references collection and manuscript drafting. DA involved in references collection and manuscript drafting. CR and PT took part in study design, study protocol, approval, revision of statistical work and manuscript. All authors have read and approved the final manuscript.

## References

[B1] Banerjee A Coronary artery disease and its problems in management J Indian Med Assoc 2001 99 474 475 12018551

[B2] WHO The World Health Report 1999. Making a difference 1999 Geneva: World Health Organization

[B3] McKeigue PM Miller GJ Marmot MG Coronary heart disease in south Asians overseas: a review J Clin Epidemiol 1989 42 597 609 10.1016/0895-4356(89)90002-4 2668448

[B4] Ko GT Chan JC Cockram CS Woo J Prediction of hypertension, diabetes, dyslipidaemia or albuminuria using simple anthropometric indexes in Hong Kong Chinese Int J Obes Relat Metab Disord 1999 23 1136 1142 10.1038/sj.ijo.0801043 10578203

[B5] Deurenberg-Yap M Chew SK Lin VF Tan BY van Staveren WA Deurenberg P Relationships between indices of obesity and its co-morbidities in multi-ethnic Singapore Int J Obes Relat Metab Disord 2001 25 1554 1562 10.1038/sj.ijo.0801739 11673781

[B6] Bhopal R Hayes L White M Unwin N Harland J Ayis S Alberti G Ethnic and socio-economic inequalities in coronary heart disease, diabetes and risk factors in Europeans and South Asians J Public Health Med 2002 24 95 105 10.1093/pubmed/24.2.95 12141592

[B7] Dudeja V Misra A Pandey RM Devina G Kumar G Vikram NK BMI does not accurately predict overweight in Asian Indians in northern India Br J Nutr 2001 86 105 112 10.1079/BJN2001382 11432771

[B8] WHO Obesity. Prevention and managing the global epidemic. Report of a WHO consultation on obesity 1998 1998 Geneva: World Health Organization WHO/NUT/NCD/98.1. 11234459

[B9] Banerji MA Faridi N Atluri R Chaiken RL Lebovitz HE Body composition, visceral fat, leptin, and insulin resistance in Asian Indian men J Clin Endocrinol Metab 1999 84 137 144 10.1210/jc.84.1.137 9920074

[B10] WHO The Asia-Pacific perspectve. Redefining obesity and its treatment. Health Communications Australia Pty.Limited. International Diabetes Institute 2000

[B11] Misra A Revisions of cutoffs of body mass index to define overweight and obesity are needed for the Asian-ethnic groups Int J Obes Relat Metab Disord 2003 27 1294 1296 10.1038/sj.ijo.0802412 14574337

[B12] Enas EASA Coronary Artery Disease in Asian Indians: An Update and Review The Internet Journal of Cardiology 2002 1

[B13] Mishra D Singh H Kuppuswamy's socioeconomic status scale--a revision Indian J Pediatr 2003 70 273 274 10.1007/BF02725598 12785303

[B14] Chobanian AV Bakris GL Black HR Cushman WC Green LA Izzo JL Jr Jones DW Materson BJ Oparil S Wright JT Jr Roccella EJ Joint National Committee on Prevention Detection Evaluation and Treatment of High Blood Pressure. National Heart Lung and Blood Institute; National High Blood Pressure Education Program Coordinating Committee Seventh Report of the Joint National Committee on Prevention, Detection, Evaluation, and Treatment of High Blood Pressure Hypertension 2003 42 6 1206 52 Epub 2003 Dec 1 10.1161/01.HYP.0000107251.49515.c2 14656957

[B15] Executive Summary of The Third Report of The National Cholesterol Education Program (NCEP) Expert Panel on Detection Evaluation And Treatment of High Blood Cholesterol In Adults (Adult Treatment Panel III) JAMA 2001 285 2486 2497 10.1001/jama.285.19.2486 11368702

[B16] American Diabetes Association. Diagnosis and Classification of diabetes mellitus Diabetes Care 2006 29 S43 48 16373932

[B17] Pezzullo J Statpages.org http://statpages.org/#Comparisons 2009

[B18] Pinto RJ Bhagwat AR Loya YS Sharma S Coronary artery disease in premenopausal Indian women: risk factors and angiographic profile Indian Heart J 1992 44 99 101 1427940

[B19] Ismail J Jafar TH Jafary FH White F Faruqui AM Chaturvedi N Risk factors for non-fatal myocardial infarction in young South Asian adults Heart 2004 90 259 263 10.1136/hrt.2003.013631 14966040 PMC1768096

[B20] Joshi P Islam S Pais P Reddy S Dorairaj P Kazmi K Pandey MR Haque S Mendis S Rangarajan S Yusuf S Risk factors for early myocardial infarction in South Asians compared with individuals in other countries JAMA 2007 297 286 294 10.1001/jama.297.3.286 17227980

[B21] Joshipura KJ Hu FB Manson JE Stampfer MJ Rimm EB Speizer FE Colditz G Ascherio A Rosner B Spiegelman D Willett WC The effect of fruit and vegetable intake on risk for coronary heart disease Ann Intern Med 2001 134 1106 1114 11412050 10.7326/0003-4819-134-12-200106190-00010

[B22] Rastogi T Vaz M Spiegelman D Reddy KS Bharathi AV Stampfer MJ Willett WC Ascherio A Physical activity and risk of coronary heart disease in India Int J Epidemiol 2004 33 759 767 10.1093/ije/dyh042 15044412

[B23] Gupta R Gupta VP Ahluwalia NS Educational status, coronary heart disease, and coronary risk factor prevalence in a rural population of India BMJ 1994 309 1332 1336 7866081 10.1136/bmj.309.6965.1332 PMC2541843

[B24] Vikram NK Pandey RM Misra A Sharma R Devi JR Khanna N Non-obese (body mass index <25 kg/m2) Asian Indians with normal waist circumference have high cardiovascular risk Nutrition 2003 19 503 509 10.1016/S0899-9007(02)01083-3 12781849

[B25] Panagiotakos DB Pitsavos C Chrysohoou C Vlismas K Skoumas Y Palliou K Stefanadis C Dietary habits mediate the relationship between socio-economic status and CVD factors among healthy adults: the ATTICA study Public Health Nutr 2008 11 1342 1349 10.1017/S1368980008002978 18616850

[B26] Tarasiuk A Greenberg-Dotan S Simon T Tal A Oksenberg A Reuveni H Low socioeconomic status is a risk factor for cardiovascular disease among adult obstructive sleep apnea syndrome patients requiring treatment Chest 2006 130 766 773 10.1378/chest.130.3.766 16963673

[B27] Higashi Y Sasaki S Nakagawa K Kimura M Noma K Hara K Matsuura H Goto C Oshima T Chayama K Yoshizumi M Low body mass index is a risk factor for impaired endothelium-dependent vasodilation in humans: role of nitric oxide and oxidative stress J Am Coll Cardiol 2003 42 256 263 10.1016/S0735-1097(03)00630-2 12875761

[B28] Rich-Edwards JW Stampfer MJ Manson JE Rosner B Hankinson SE Colditz GA Willett WC Hennekens CH Birth weight and risk of cardiovascular disease in a cohort of women followed up since 1976 BMJ 1997 315 396 400 9277603 10.1136/bmj.315.7105.396 PMC2127275

[B29] Tanis BC Kapiteijn K Hage RM Rosendaal FR Helmerhorst FM Dutch women with a low birth weight have an increased risk of myocardial infarction later in life: a case control study Reprod Health 2005 2 1 10.1186/1742-4755-2-1 15642119 PMC548672

[B30] Pais P Pogue J Gerstein H Zachariah E Savitha D Jayprakash S Nayak PR Yusuf S Risk factors for acute myocardial infarction in Indians: a case-control study Lancet 1996 348 358 363 10.1016/S0140-6736(96)02507-X 8709733

[B31] Choudhury L Marsh JD Myocardial infarction in young patients Am J Med 1999 107 254 261 10.1016/S0002-9343(99)00218-1 10492319

[B32] Dietrich M Block G Norkus EP Hudes M Traber MG Cross CE Packer L Smoking and exposure to environmental tobacco smoke decrease some plasma antioxidants and increase gamma-tocopherol in vivo after adjustment for dietary antioxidant intakes Am J Clin Nutr 2003 77 160 166 12499336 10.1093/ajcn/77.1.160

[B33] Chopra M O'Neill ME Keogh N Wortley G Southon S Thurnham DI Influence of increased fruit and vegetable intake on plasma and lipoprotein carotenoids and LDL oxidation in smokers and nonsmokers Clin Chem 2000 46 1818 1829 11067818

[B34] National Institute of Nutrition: 25 years of National Nutrition Monitoring Bureau Indian Council of Medical Research. Hyderabad India 1997

[B35] Molarius A Seidell JC Differences in the association between smoking and relative body weight by level of education Int J Obes Relat Metab Disord 1997 21 189 196 10.1038/sj.ijo.0800386 9080257

[B36] Juutilainen A Lehto S Ronnemaa T Pyorala K Laakso M Type 2 diabetes as a "coronary heart disease equivalent": an 18-year prospective population-based study in Finnish subjects Diabetes Care 2005 28 2901 2907 10.2337/diacare.28.12.2901 16306552

[B37] Abate N Chandalia M Ethnicity and type 2 diabetes: focus on Asian Indians J Diabetes Complications 2001 15 320 327 10.1016/S1056-8727(01)00161-1 11711326

[B38] Ramachandran A Snehalatha C Kapur A Vijay V Mohan V Das AK Rao PV Yajnik CS Prasanna Kumar KM Nair JD High prevalence of diabetes and impaired glucose tolerance in India: National Urban Diabetes Survey Diabetologia 2001 44 1094 1101 10.1007/s001250100627 11596662

[B39] Khaw KT Wareham N Luben R Bingham S Oakes S Welch A Day N Glycated haemoglobin, diabetes, and mortality in men in Norfolk cohort of european prospective investigation of cancer and nutrition (EPIC-Norfolk) BMJ 2001 322 15 18 10.1136/bmj.322.7277.15 11141143 PMC26599

[B40] O'Keefe JH Jr Miles JM Harris WH Moe RM McCallister BD Improving the adverse cardiovascular prognosis of type 2 diabetes Mayo Clin Proc 1999 74 171 180 10.4065/74.2.171 10069357

[B41] Radhika G Sathya RM Sudha V Ganesan A Mohan V Dietary salt intake and hypertension in an urban south Indian population--[CURES - 53] J Assoc Physicians India 2007 55 405 411 17879493

[B42] Hooper LBC Davey Smith G Review: restricted dietary salt intake can reduce blood pressure but does not reduce death or cardiovascular morbidity Evidence-Based Medicine 2003 8 139 10.1136/ebm.8.5.139

[B43] Huang Z Willett WC Manson JE Rosner B Stampfer MJ Speizer FE Colditz GA Body weight, weight change, and risk for hypertension in women Ann Intern Med 1998 128 81 88 9441586 10.7326/0003-4819-128-2-199801150-00001

